# Selective identification of somatic mutations in pancreatic cancer cells through a combination of next-generation sequencing of plasma DNA using molecular barcodes and a bioinformatic variant filter

**DOI:** 10.1371/journal.pone.0192611

**Published:** 2018-02-16

**Authors:** Yoji Kukita, Kazuyoshi Ohkawa, Ryoji Takada, Hiroyuki Uehara, Kazuhiro Katayama, Kikuya Kato

**Affiliations:** 1 Department of Molecular and Medical Genetics, Research Institute, Osaka Medical Center for Cancer and Cardiovascular Diseases, Osaka, Japan; 2 Department of Hepatobiliary and Pancreatic Oncology, Osaka Medical Center for Cancer and Cardiovascular Diseases, Osaka, Japan; CNR, ITALY

## Abstract

The accuracy of next-generation sequencing (NGS) for detecting tumor-specific mutations in plasma DNA is hindered by errors introduced during PCR/sequencing, base substitutions caused by DNA damage, and pre-existing mutations in normal cells that are present at a low frequency. Here, we performed NGS of genes related to pancreatic cancer (comprising 2.8 kb of genomic DNA) in plasma DNA (average 4.5 ng) using molecular barcodes. The average number of sequenced molecules was 900, and the sequencing depth per molecule was 100 or more. We also developed a bioinformatic variant filter, called CV78, to remove variants that were not considered to be tumor-specific, i.e., those that are either absent or occur at low frequencies in the Catalogue of Somatic Mutations in Cancer database. In a cohort comprising 57 pancreatic cancer patients and 12 healthy individuals, sequencing initially identified variants in 31 (54%) and 5 (42%), respectively, whereas after applying the CV78 filter, 19 (33%) and zero were variant-positive. In a validation cohort consisting of 86 patients with pancreatic cancer and 20 patients with intraductal papillary mucinous neoplasm (IPMN), 62 (72%) with pancreatic cancer patients and 10 (50%) IPMN patients were initially variant positive. After CV78 filtering, these values were reduced to 32 (37%) and 1 (5%), respectively. The variant allele frequency of filtered variants in plasma ranged from 0.25% to 76.1%. Therefore, combining NGS and molecular barcodes with subsequent filtering is likely to eliminate most non-tumor-specific mutations.

## Introduction

Circulating tumor DNA (ctDNA) is cell-free DNA (cfDNA) that is released from dying/dead cancer cells into the blood stream. It is a biomarker of cancer and is expected to have wide applications, such as the early detection of cancer and monitoring of drug resistance [1]. However, some characteristics of ctDNA make it poorly suited for use in diagnostic applications. One milliliter of blood contains cfDNA from one to several thousand genomes as fragments with an average size of 170 base pairs. The rare mutations from cancer cells must be detected among the vast amount of DNA from normal cells and quantitated.

Digital PCR [2] and next-generation sequencing (NGS) are becoming the technologies of choice for detecting cancer variants. However, the high sequencing error rate of current NGS platforms is a major problem. In addition, sequencing multiple sites or genomic regions dramatically increases the number of false positives. The introduction of molecular barcodes [3–7] is likely to solve this problem. So called “barcoding” involves the labeling of DNA fragments with unique oligonucleotides, typically 10–15 bases long, which enables reads from the same DNA fragment to be grouped together. Constructing a consensus read from the grouped reads eliminates sequencing errors as well as errors introduced during PCR. However, molecular barcodes cannot detect base substitutions in genomic DNA introduced by DNA damage, and somatic mutations that preexist at a low frequency in the cells of healthy individuals [8] also make it difficult to discriminate ctDNA from cfDNA originating from normal cells. Thus, to use NGS for diagnostic purposes, such variants must be removed.

We previously developed a high-fidelity sequencing method that utilizes molecular barcodes called the non-overlapping integrated read sequencing system (NOIR-SS) [4]. NOIR-SS is distinct from other methods that utilize molecular barcodes owing to its ability for absolute quantitation of cancer mutations. Since a substantial fraction (or majority) of barcode tags include PCR/sequencing errors, removal of erroneous barcode tags is crucial for absolute quantitation of cancer mutations. NOIR-SS removes erroneous barcode tags during data analysis.

Since surgical resection of localized tumors improves overall survival, early detection of pancreatic cancer would have substantial health benefits [9]. For early detection, high specificity is especially important. In this study, we devised a NOIR-SS-based assay for a panel of genes related to pancreatic cancer. In the first step, variants, including those present in healthy individuals, are identified. Therefore, we developed a bioinformatic filter to remove variants that are unlikely to be somatic mutations in cancer tissues. The results showed that the filter completely removed such inappropriate variants. The performance of the assay and filter system was validated using an independent cohort of patients with intraductal papillary mucinous neoplasm (IPMN) and pancreatic cancer. Sequencing based on molecular barcodes along with the filter we developed is likely to eliminate most variants not specific to cancer cells, while maintaining sensitivity comparable to that of conventional digital PCR/deep sequencing.

## Materials and methods

### Patients and samples

Blood samples were obtained from patients with pancreatic cancer or IPMN between January 2012 and February 2016 at the Osaka Medical Center for Cancer and Cardiovascular Diseases. Plasma preparation and DNA extraction were performed as described previously [10]. Tissue samples were obtained using endoscopic ultrasound-guided fine-needle aspiration. Written informed consent was obtained from all the patients. This study was approved by the ethics committee of the O Osaka Medical Center for Cancer and Cardiovascular Diseases.

#### Adapters and primers for amplification of target regions

The targeted regions of genes related to pancreatic cancer are shown in S1 Table. The sequences of the adapters and primers are shown in S2 Table. A 30-base-long adapter sequence, including the primer sequence for Ion Torrent sequencing, was joined to a 5-base sequence for indexing individuals, a 12-base sequence for indexing molecules, and a 20-base spacer at the 3ʹ end.

### Library construction with barcoded strands

Library construction for NOIR-SS was performed according to a modification of a previously described procedure [4]. For each plasma sample, we prepared two separate reaction mixtures with two gene-specific primers (shown with a “1” or “2” suffix in S2 Table) since our PCR system did not allow the use of primer pairs. Cell-free DNA obtained from approximately 1 ml of whole blood (average 4.5 ng, median 3.1 ng).was end-repaired in a 15 μL reaction containing 50 mM Tris-HCl, pH 8.0, 10 mM MgCl_2_, 10 mM dithiothreitol, 1 mM ATP, 0.4 mM dNTPs, 2.4 units of T4 DNA polymerase (Takara Bio, Kusatu, Japan), 7.5 units of T4 polynucleotide kinase (NEB, Ipswich, MA, USA), and 0.5 units of KOD DNA polymerase (Toyobo, Osaka, Japan), which was incubated for 30 minutes at 25°C and then for 20 minutes at 75°C. Adapters tagged with the 12-nucleotide barcode sequence were ligated in a 20 μL end-repair reaction containing 0.5 μL of 10× T4 DNA ligase buffer (NEB), 40 pmol of adapter, and 2000 units of T4 DNA ligase (NEB), which was incubated at 25°C for 15 minutes. The ligation products were purified twice with 1.2× volumes of AMPure XP beads (Beckman Coulter, Brea, CA, USA). The purification beads were then mixed with 20 μL of the linear amplification reaction mix (1× Q5 Reaction Buffer [NEB], 0.2 mM dNTPs, 6 μM gene-specific primer mix [suffix “1” or “2” in S2 Table], and 0.4 units of Q5 Hot Start High-Fidelity DNA Polymerase [NEB]). After the AMPure XP beads were removed, the amplification was performed in the reaction: denaturation at 98°C for 30 seconds, and then 15 cycles of 10 seconds at 98°C and 2 minutes at 65°C. Then, 1.2 μL of 100 μM T_PCR_A was added to the reaction, and the mixture was incubated as follows: 15 cycles of 10 seconds at 98°C, 30 seconds at 65°C, and 30 seconds at 72°C. The amplification products were purified once with 1.2× volumes of AMPure XP, and recovered in 20 μL of 0.1× TE. Three microliters of the purified products were added to two tubes containing PCR amplification solution (20 μL each): 1× High Fidelity PCR Buffer (Thermo Fisher Scientific, Waltham, MA, USA), 0.2 mM dNTPs, 2 mM MgSO_4_, 0.5 μM T_PCR_A, 0.5 μM nested-primer-mix (suffix “L” or “H” in S2 Table), and 0.4 units of Platinum Taq DNA Polymerase, High Fidelity (Thermo Fisher Scientific). The thermal cycling program was as follows: 2 minutes of denaturation at 95°C followed by 25 (nested-primer-mix with suffix “H”) or 30 (nested-primer-mix with suffix “L”) cycles of 15 seconds at 95°C and 1 minute at 63°C. The amplification products were purified with 1.2× volumes of AMPure XP beads. The product concentration was determined using the Qubit dsDNA HS Assay Kit or Quant-iT PicoGreen dsDNA Assay Kit (Thermo Fisher Scientific).

### Sequencing and data analysis

Massively parallel sequencing was performed using an Ion Torrent Proton sequencer (Thermo Fisher Scientific) according to the manufacturer’s protocol. Torrent Suite (Thermo Fisher Scientific) was used to convert the raw data into base calls and to extract the FASTQ files of the sequence reads.

Reads in FASTQ format were sorted using the 5-base indices for the assignment of individuals. The sequences between the 5-base indices and spacer sequences were obtained and used as molecular barcode tags. Reads with a total length, including the spacer and the sequence following it, >50 bases were aligned to target regions using BWA-MEM [11], whereas reads with short mapped ends (<40 bases) were discarded. Reads with the same barcode sequences were grouped together; erroneous barcode tags were detected and removed as described previously [4]. Consensus sequences for reads with the same barcode were created using VarScan [12] as described previously [4]. When more than 85% of reads had the same base at a position, this base was selected as the consensus. For variant detection, we applied a Poisson distribution model to calculate the sequencing error as previously described [4]. We evaluated each target region for the presence of variant(s), setting *P* = 10^−4^ as the threshold for detection. We evaluated each base position in codons 12 and 13 of the *KRAS* gene at the specified threshold value [10]. Common SNP sites and error-prone sites were not considered in our analysis. We used Genome Reference Consortium human genome build 37 (GRCh37/hg19) as the reference genome.

Deep sequencing data were analyzed as described previously [10], except that the threshold was set at 0.11% (1/900, where the denominator refers to the average number of molecules sequenced per target region).

## Results

### NOIR-SS for pancreatic cancer

**Fig 1:**
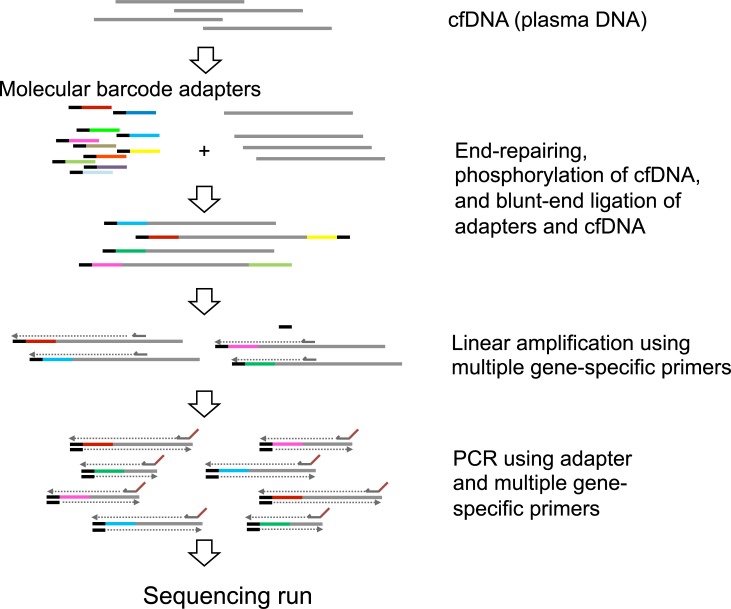
Construction of the molecular barcode library, generated using a revised version of non-overlapping integrated read sequencing system (NOIR-SS). Cell-free DNA is end-repaired, phosphorylated and ligated to adapters including barcode sequences. DNA fragments with the adapters are subjected to linear amplification using multiple gene-specific primers. To construct sequencing libraries, the products are subjected to PCR using primers including sequences indispensable for the Ion Torrent sequencer.

We used NOIR-SS to target regions of eight pancreatic cancer-related genes: *KRAS*, *TP53*, *SMAD4*, *CTNNB1*, *CDKN2A*, *GNAS*, *HRAS*, and *NRAS*. The total size of the target regions was 2.8 kb. In the original version of NOIR-SS [4], barcode-tagged adapters were attached to the template DNA molecules after digestion with restriction enzymes. Since this procedure limited the choice of the target regions, here we attached the barcode-tagged adapters directly to the undigested ends of the cfDNA. As end positions of cfDNAs were variable, target sizes ranged from 110 to 260 bp, including adapter sequences. For library construction, we amplified target regions with approximately 4.5 ng of cell-free DNA using the adapter and a mixture of gene-specific primers after a linear amplification step with only the gene-specific primers. The reaction scheme is presented in Fig 1. The library was subjected to NGS on an Ion Torrent sequencer. Sequence reads were grouped using molecular barcodes. After removal of erroneous sequences, high quality sequence data were used to build a consensus sequence for each read group. The sequencing depth was at least 100 per molecule. The average number of sequenced molecules was 900 per target region.

### Construction of a filter to remove non tumor-specific variants

The first data set was obtained from a cohort of 12 healthy individuals and 57 patients with pancreatic cancer. The variant detection results are summarized in the top-half of Table 1. In the samples from healthy individuals, 12 variants were detected (S3 Table). Although variants present in less than 1% of the reads do not affect the analysis in conventional NGS applications, such as whole genome or exome sequencing, they are a serious problem in the detection of ctDNA. In our case, 5 out of 12 healthy individuals were variant-positive (Table 1, top right). Therefore, direct use of variants identified by NOIR-SS is not appropriate for the diagnosis of pancreatic cancer. Therefore, we developed a filter to remove variants present in healthy individuals. To develop our variant filter, we used the first set of 12 healthy individuals and 57 patients with pancreatic cancer as “training data,” as is the norm when solving classification problems in the field of statistics.

**Table 1:** Variants in pancreatic cancer identified by NOIR-SS and the CV78 filter.

	Number of variants (before filtering)	Number of variants (after filtering)	Recovery rate (%)	Total number of patients	Variant-positive patients (before filtering)	(%)	Variant-positive patients (after filtering)	(%)	Average number of variants per variant-positive patient (before filtering)	Average number of variants per variant-positive patient (after filtering)
First set										
Healthy individuals	12	0	0.0	12	5 (0)	41.7	0 (0)	0.0	2.40	N/A
Pancreatic cancer, localized	27	5	18.5	32	15 (4)	46.9	4 (2)	12.5	1.80	1.25
Pancreatic cancer, metastatic	42	27	64.3	25	16 (13)	64.0	15 (12)	60.0	2.63	1.80
Pancreatic cancer, all	69	32	46.4	57	31 (17)	54.4	19 (14)	33.3	2.23	1.68
Second set										
IPMN	12	1	8.3	20	10 (3)	50.0	1 (0)	5.0	1.20	1.00
Pancreatic cancer, localized	57	17	29.8	45	30 (7)	66.7	12 (7)	26.7	1.90	1.42
Pancreatic cancer, metastatic	79	36	45.6	41	32 (19)	78.0	20 (16)	48.8	2.47	1.80
Pancreatic cancer, all	136	53	39.0	86	62 (26)	72.1	32 (23)	37.2	2.19	1.66

The numbers of KRAS variants are shown in parentheses. Two IPMN cases showed malignant histology (stage I). After filtering refers to the variants that remained after the CV78 filter was applied.

All known cancer-specific mutations are stored in a public database called the Catalogue of Somatic Mutations in Cancer (COSMIC) [13]. Recent large-scale efforts to characterize cancer genomes, such as the international cancer genome consortium [14] and the cancer genome atlas [15], are assumed to have identified most mutations originating in primary tumors. However, 10 of the 12 variants identified in the healthy individuals were not cataloged in COSMIC. Tumor-specific mutations have biological activities, and their base positions should be restricted to hot spots. On the contrary, non-functional variants are likely to occur randomly. To handle this, we made the following two assumptions: 1) COSMIC includes all somatic mutations present in cancer tissues; 2) low-frequency entries in COSMIC may arise from artifacts such as DNA damage or PCR/sequencing errors. A recent report supports the appropriateness of the second assumption [16]. Accordingly, we removed variants not cataloged in COSMIC (version 78) and single-entry variants, except variants in *TP53*. Since a larger number of somatic mutations have been cataloged for the coding region of *TP53*, a more stringent criterion was appropriate. Accordingly, we removed *TP53* variants with less than 10 entries. Thus, of the 16 *TP53* variants identified in patients with pancreatic cancer in the first data set, we removed five variants with no entry, one with a single entry, and three with 2–9 entries in COSMIC. This bioinformatic process was designated as the CV78 filter. The CV78 filter removed all variants present in the healthy individuals (Table 1, top). The sensitivity of the assay to detect pancreatic cancer patients, namely the fraction of variant-positive cancer patients, was decreased from 54.4% to 33.3% with the CV78 filter. Since insertion/deletion errors were uncommon in our results (one insertion and one deletion), we excluded them from the analysis.

For 10 patients with pancreatic cancer, both plasma and tumor samples were available. Their variants identified with NOIR-SS are shown in Table 2. Among the 35 variants identified, six were only detected in plasma samples, and all six of these variants were removed by the CV78 filter. To examine whether these variants were derived from leucocytes, we sequenced the corresponding target regions of leucocyte DNA. As shown in S4 Table, no variants were found in leucocytes. As variants in cfDNA are derived from dead/dying cells, it may be difficult to deduce those sequences from living cells. The CV78 filter also discriminated variants present in normal cells from those present in tumor cells. However, it should be noted that six variants present in the tumor samples were also removed by the CV78 filter, indicating the possible loss of true mutations. Except for variants identified in the sample from patient P188, the frequencies of all variant alleles were low. This indicates that these variants do not represent genetic abnormalities present in the majority of the cell population.

**Table 2:** Comparison of variants detected in plasma and tumor tissue from 10 patients with pancreatic cancer.

Gene	Chromosome position	Amino acid position (base)	Base change	Variant fraction (tumor tissue)	Variant fraction (plasma)	CV78 filter	plasma only
Patient P159							
*KRAS*	chr12:25398284	12 (2)	G > A	11.6	-	Pass	
*SMAD4*	chr18:48575209	135 (1)	C > T	16.6	-	Pass	
*TP53*	chr17:7573988	347 (1)	G > T	0.7	-	No	
Patient P162							
*KRAS*	chr12:25398284	12 (2)	G > A	40.3	19.3	Pass	
*KRAS*	chr12:25398283	12 (3)	T > G	40.4	20.0	Pass	
*SMAD4*	chr18:48604701	508 (2)	G > A	26.4	10.1	Pass	
Patient P166							
*KRAS*	chr12:25398284	12 (2)	G > A	44.0	34.1	Pass	
*TP53*	chr17:7579365	108 (1)	G > T	-	0.4	No	+
*TP53*	chr17:7577120	273 (2)	G > A	26.5	19.2	Pass	
Patient P177							
*CDKN2A*	chr9:21971179	60 (2)	C > T	-	0.5	No	+
*HRAS*	chr11:533854	68 (1)	C > T	-	0.4	No	+
*KRAS*	chr12:25398284	12 (2)	G > T	1.2	-	Pass	
*SMAD4*	chr18:48603094	465 (3)	G > T	0.7	-	No	
*TP53*	chr17:7578231	206 (3)	G > T	0.9	-	No	
Patient P179							
*CDKN2A*	chr9:21971120	80 (1)	C > T	7.9	-	Pass	
*KRAS*	chr12:25398285	12 (1)	G > T	41.1	0.5	Pass	
*SMAD4*	chr18:48591903	356 (1)	C > A	2.4	-	No	
*SMAD4*	chr18:48603032	445 (1)	C > T	4.3	0.8	Pass	
*TP53*	chr17:7578272	193 (1)	C > G	38.7	-	Pass	
Patient P183							
*KRAS*	chr12:25398284	12 (2)	G > A	42.6	9.1	Pass	
Patient P184							
*CDKN2A*	chr9:21971096	88 (1)	G > T	23.4	-	Pass	
*KRAS*	chr12:25398285	12 (1)	G > C	37.2	-	Pass	
*SMAD4*	chr18:48604734	519 (2)	A > T	-	0.5	No	+
*TP53*	chr17:7577545	246 (1)	A > G	56.3	-	Pass	
Patient P186							
*CDKN2A*	chr9:21971120	80 (1)	C > T	2.2	-	Pass	
*KRAS*	chr12:25398284	12 (2)	G > A	12.9	0.5	Pass	
*TP53*	chr17:7578406	175 (2)	G > A	11.9	0.7	Pass	
Patient P187							
*KRAS*	chr12:25398284	12 (2)	G > T	17.2	0.2	Pass	
*KRAS*	chr12:25398285	12 (1)	G > A	17.0	0.2	Pass	
*SMAD4*	chr18:48603033	445 (2)	G > A	-	0.4	No	+
Patient P188							
*GNAS*	chr20:57484420	201 (1)	C > T	27.7	-	Pass	
*KRAS*	chr12:25398291	10 (1)	G > T	-	1.8	No	+
*KRAS*	chr12:25398285	12 (1)	G > T	41.9	-	Pass	
*KRAS*	chr12:25380301	53 (1)	T > A	18.4	-	No	
*KRAS*	chr12:25380300	53 (2)	T > A	18.6	-	No	

The variant fractions are presented as percentages.

The prevalence of ctDNA is higher in metastatic cancer than in localized cancer [17]. This characteristic was obscured in the initial analysis, but was apparent after the filtering procedure.

### Differentiation of IPMN and pancreatic cancer

The entire process, i.e., the identification of variants with NOIR-SS and subsequent filtering of variants for cancer-specificity, was validated on an independent sample set. We designated it as the second sample set, and it included plasma samples from 20 patients with IPMN and 86 patients with pancreatic cancer. Plasma samples from patients with pancreatic cancer included in the second set were obtained later than those included in the first data set, except for the plasma samples with paired tissue samples. All the samples in the second set were assayed and analyzed only after development of the CV78 filter.

IPMN is a benign neoplasm that grows within the pancreatic duct, and therefore is unlikely to release cfDNA into the blood stream. *KRAS* mutations are rarely detected in the plasma of patients with benign neoplasms [18]. Since a significant proportion of IPMN cases progress to pancreatic cancer [19], differentiation of IPMN from pancreatic cancer may have substantial clinical benefits.

Initially, 10 out of 20 patients with IPMN were variant-positive; however, after CV78 filtering, only one patient was variant-positive (the bottom section of Table 1). In contrast, 32 out of 86 patients with pancreatic cancer were variant-positive after CV78 filtering. The CV78 filter decreased the assay sensitivity from 72.1% to 37.2%; the relative reduction in assay sensitivity was similar to that of the first data set. Use of the filter increased the identification of variants present in metastatic cancer, which would have otherwise remained obscure. The major characteristics of the first sample set were reproduced by the second sample set.

### Other characteristics of variants identified by NOIR-SS

Variants identified by NOIR-SS were classified according to their presence within specific genes (Table 3). Filtered variants were defined as those selected by the CV78 filter. A characteristic common to the first and the second sample sets was the high recovery rate of *KRAS* mutations with the CV78 filter. This is due to mutational hotspots present within codons 12 and 13. There was no significant difference in the recovery rates (i.e., the percentage of selected variants) between the first and the second sample sets.

**Table 3:** Variants classified in each gene.

	Variants (first set)	Filtered variants (first set)	Recovery rate (%)	Variants (second set)	Filtered variants (second set)	Recovery rate (%)
*CDKN2A*	2	1	N/A	9	2	22.2
*CTNNB1*	2	0	N/A	0	0	N/A
*GNAS*	1	1	N/A	6	2	33.3
*HRAS*	6	1	16.7	14	1	7.1
*KRAS*	20	17	85.0	28	25	89.3
*NRAS*	6	0	0.0	2	1	N/A
*SMAD4*	16	4	25.0	17	5	29.4
*TP53*	16	8	50.0	60	17	28.3
Total	69	32	46.4	136	53	39.0

N/A: The recovery rate was not calculated when the number of variants was five or less.

**Fig 2:**
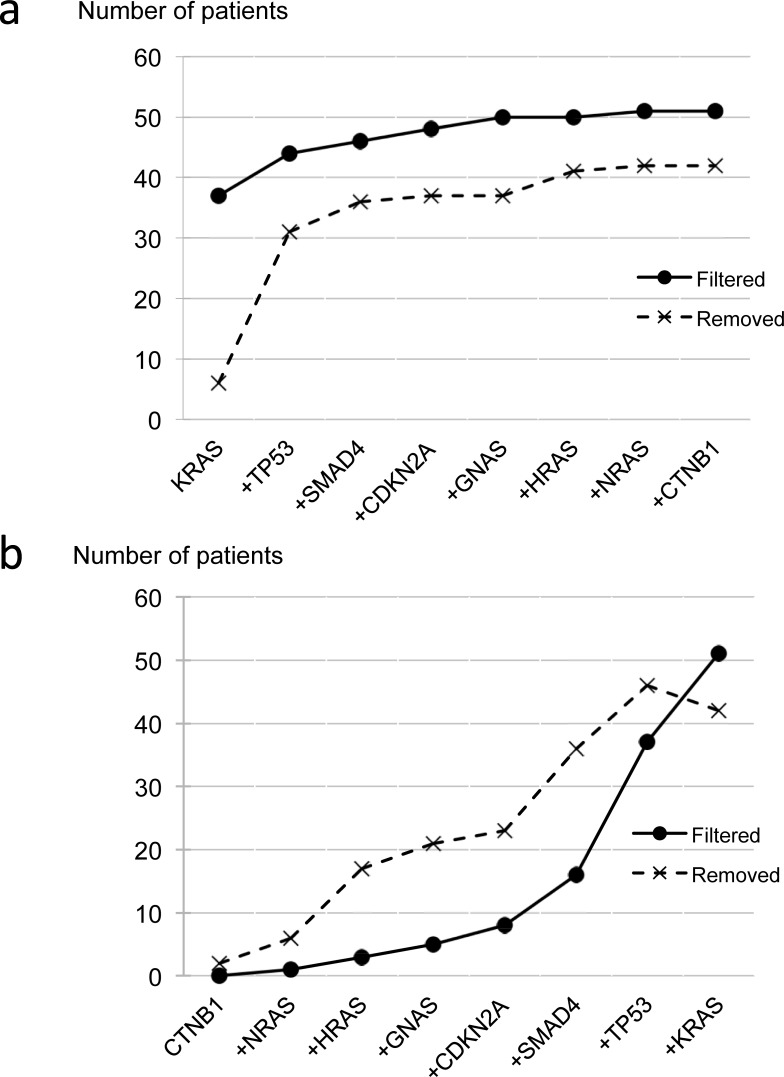
Cumulative numbers of variant-positive patients obtained by addition of genes. A, Genes are in descending order according to the number of variants. B, Genes are in ascending order according to the number of variants. The y-axis shows the number of patients, and the x-axis shows genes in order of the number of variant-positive patients identified. Solid line: patients identified by filtered variants; broken line: patients removed by CV78 filtering.

Since there was no significant difference between the results from the first and second sample sets, subsequent analyses were performed by combining data from both sample sets. To examine the contribution of individual genes (after application of the CV78 filter), we plotted the cumulative number of variant-positive patients identified for each gene (Fig 2). In Fig 2A, the genes are arranged along the x-axis in descending order of the number of variant-positive patients identified. Conversely, in Fig 2B, the genes are arranged along the x-axis in descending order of the number of variant-positive patients identified per gene. Fig 2A shows that the highest number of variant-positive patients had variants in *KRAS* (explained below). In addition, only a small number of variant-positive patients was removed after applying the CV78 filter. Fig 2B shows that the numbers of selected and removed variant-positive patients increased as the number of genes increased. Using variants in all genes except *KRAS* identified the same number of filtered variant-positive patients as variants in *KRAS* only, and the numbers of variant-positive patients after filtering and their proportions among the total number of patients (143) were as follows: all genes, 51 (35.7%); *KRAS*, 37 (25.9%); other genes excluding *KRAS*, 37 (25.9%).

A statistical analysis of the base substitution patterns is shown in S5 Table. Among the filtered variants, 23.3% were G>T/C>A transversions and 62.8% were C>T/G>A transitions. Among the variants removed by the CV78 filter, 32.2% were G>T/C>A transversions and 40.6% were C>T/G>A transitions. These transversions and transitions were the major mutations detected in both data sets. Their dominance among the filtered variants suggests that tumor-specific mutations likely arise via oxidation/deamination and are repaired by cell proliferation.

**Fig 3:**
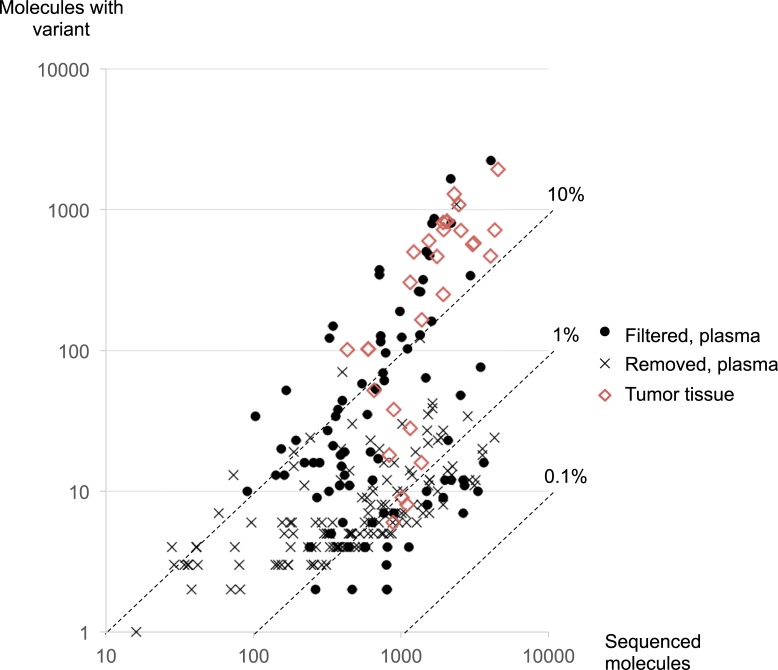
Distribution of the sequenced molecules. The x-axis shows the number of sequenced molecules, and the y-axis shows the number of molecules in which a variant was detected. The diagonal broken lines indicate variant allele frequencies. Closed circles: filtered variants; Cross marks: variants removed by CV78 filtering; Diamonds: variants in tumor tissue.

All samples were displayed on a scatter plot, with the number of sequenced molecules on the x-axis and number of variant molecules on the y-axis (Fig 3). Variant allele frequencies are indicated by broken diagonal lines. The variant allele frequency of filtered and removed variants ranged from 0.25–76.1% and from 0.35–45.9%, respectively. However, the distribution patterns between the two types of variants were different. The variant allele frequency of filtered variants varied widely in their respective proportions, from more than 10% to less than 1% of sequenced molecules. In contrast, the removed variants rarely made up more than 10% of all sequenced molecules, and most were approximately 1%. More than two-thirds of variants isolated from tissue samples showed high variant allele frequency.

Finally, we compared the performance of the method developed in this study to that of conventional deep sequencing. Since the presence of *KRAS* mutations is used as the standard for identifying ctDNA in patients with pancreatic cancer, we compared the ability of our method to detect *KRAS* mutations to that of conventional deep sequencing. In the first analysis, we analyzed our sequence data without using the molecular barcodes, thus regarding them as conventional deep sequencing data. In the second analysis, we utilized the data from a large-scale case-control study of a population of European descent by Le Calvez-Kelm et al. [18] conducted using deep sequencing. Sensitivity and specificity were respectively defined as the proportion of *KRAS* mutation-positive samples among samples from patients of pancreatic cancer and the proportion of *KRAS* mutation-negative samples among samples from the control population. The control population in the first analysis included healthy individuals as well as IPMN patients. The respective calculated sensitivity and specificity were as follows: NOIR-SS/CV78 (our data), 25.9% and 100%; deep sequencing (our data), 30.8% and 96.9%; and deep sequencing (data from Le Calvez-Kelm et al.), 20.9% and 96.3%. Two-by-two tables and representative parameters are shown in S6 Table. In comparison, the sensitivity of two previous studies that used droplet digital PCR [20, 21] was 32.0%. These two studies did not report the specificity of their method using control populations.

## Discussion

Molecular barcode technology eliminates PCR/sequencing errors and generally achieves an error rate of approximately 10^−5^, regardless of platform and assay conditions [3–5]. Thus, this technology is likely to be indispensable for NGS of cfDNA. However, it is not effective for removing artifacts introduced before PCR, such as those introduced by damage to genomic DNA during or before sample preparation. In addition, preexisting somatic mutations in normal cells [8] make it difficult to discriminate ctDNA from cfDNA originating from normal cells. Current knowledge regarding the prevalence of mutations in nuclear DNA is based on living cells, and comparatively little is known about mutations in DNA entering the circulation from dead/dying cells. Somatic mutations in normal cells might become more frequent just before death, especially under stress conditions, such as cancer.

We identified a number of variants in healthy individuals and patients with pancreatic cancer using NOIR-SS. Then, we applied a bioinformatic filter, called the CV78 filter, to select ctDNA variants based on the COSMIC database. Variants with numbers of entries exceeding a preset threshold were selected and regarded as somatic mutations specific to the tumor tissue; the other variants were discarded. We achieved near-complete elimination of variants present in control samples, and complete elimination of variants not present in tumor samples. The combined application of NOIR-SS and the CV78 filter is likely to successfully identify somatic mutations specific to ctDNA. Based on the evidence presented here, we support conducting a larger study for confirmation.

One of the benefits of using ctDNA as a biomarker is that it allows tracking of new mutations potentially involved in disease progression and/or drug resistance in metastatic lesions [22]. Such mutations may not be in the COSMIC database because of the difficulty in sampling metastatic lesions. However, it is important to note that our approach is diagnostic, and therefore is not appropriate for the research described in this paragraph.

The CV78 filter was calibrated to remove all non-specific variants. It removed all low-frequency variants, irrespective of whether they were a genuine mutation or an error. As a result, a few of the somatic mutations detected in tumor tissues were also removed by the CV78 filter. Thus, the current filter thresholds might be too stringent, and there is some room for improvement. A different approach for evaluating whether a variant is a mutation or an error would involve referencing normal DNA sequences deposited in in-house databases [5, 23]. The integrated digital error suppression (iDES) technique includes such a bioinformatic step [5]. Since iDES is not based on previous knowledge of tumor-specific mutations, it is suitable for exploratory studies. Although the iDES identifies errors due to DNA damage to some extent, it by itself, cannot remove mutations present in normal cells.

Most techniques, including NOIR-SS, cannot discriminate true mutations from variants introduced by DNA damage. In contrast, duplex sequencing [6] can discriminate variants present in both strands (mutations) of the DNA and those present in only a single strand (DNA damage). Therefore, comprehensive analysis of cfDNA from patients with cancer using duplex sequencing would be highly beneficial for understanding the origin of variants. However, duplex sequencing is not suitable for diagnostic use, because it requires a vast amount of DNA [5]. Generally, it is more important to discriminate mutations occurring in the tumor tissue from mutations occurring in normal tissue, than it is to discriminate between genuine mutations and DNA damage.

This study demonstrated the benefit of using NOIR-SS and the CV78 filter for the detection of somatic mutations specific to ctDNA. However, the sensitivity of the assay does not suffice for early detection. We extracted plasma DNA from 1 mL whole blood samples for each sequencing reaction; therefore, the sample volume could be increased. Increasing the yield of plasma DNA may be especially beneficial for NOIR-SS, as the sensitivity of detection increases as the number of sequenced molecules increases. As pointed out previously [18], the low levels of ctDNA prevent early diagnosis of cancer. We should continue to improve our assay technique to overcome this limitation and to detect all molecules present in plasma.

## Supporting information

S1 Table Target regions in pancreatic cancer-related genes. (XLSX)

S2 Table Sequences of adapters and primers. (XLSX)

S3 Table Variants identified in samples from healthy individuals. (XLSX)

S4 Table Detailed analysis of variants only appeared in plasma presented in Table 2. (XLSX)

S5 Table Substitution patterns in filtered and removed variants. (XLSX)

S6 Table Two-by-two tables and representative parameters for comparison of different sequencing/analysis methods. (XLSX)

S7 Table The comprehensive list of all variants. (XLSX)
